# Suicidal behaviours among Ugandan university students: a cross-sectional study

**DOI:** 10.1186/s12888-022-03858-7

**Published:** 2022-04-01

**Authors:** Mark Mohan Kaggwa, Innocent Arinaitwe, Moses Muwanguzi, Elicana Nduhuura, Jonathan Kajjimu, Moses Kule, Sarah Maria Najjuka, Rahel Nkola, Noble Ajuna, Nicholas Kisaakye Wamala, Ivan Machacha, Mohammed A. Mamun, Cyrus Su-Hui Ho, Mark D. Griffiths, Godfrey Zari Rukundo

**Affiliations:** 1 Department of Psychiatry, Faculty of Medicine, Mbarara University of Science and Technology, Mbarara, 1410, Uganda; 2 African Centre for Suicide Prevention and Research, Mbarara, 379, Uganda; 3 Faculty of Medicine, Mbarara University of Science and Technology, Mbarara, 1410, Uganda; 4 Department of Psychiatry, Mbarara Regional Referral Hospital, Mbarara, 40, Uganda; 5 College of Health Sciences, Makerere University, Kampala, 7072, Uganda; 6 Department of Nursing, Bishop Stuart University, Mbarara, Uganda; 7 Faculty of Clinical Medicine and Dentistry, Kampala International University – western Campus, Kampala, 20000, Uganda; 8 School of Medicine, Kabale University, Kabale, 364, Uganda; 9 CHINTA Research Bangladesh, Savar, Dhaka 1342 Bangladesh; 10 Department of Public Health and Informatics, Jahangirnagar University, Savar, Dhaka 1342 Bangladesh; 11 Department of Public Health, Daffodil International University, Dhaka, Bangladesh; 12 Department of Psychological Medicine, National University of Singapore, Singapore, 119007 Singapore; 13 Psychology Department, Nottingham Trent University, 50 Shakespeare Street, Nottingham, NG1 4FQ UK

**Keywords:** Suicide, University students, University tuition fees, COVID-19, Chronic physical medical conditions, Uganda

## Abstract

**Background:**

Suicide remains the leading cause of death among university students often resulting from multiple physical and psychological challenges. Moreover, suicidal behaviours among students appear to have increased due to the COVID-19 pandemic according to some studies.

**Objective:**

To explore the prevalence and associated factors for suicidal ideation, suicide plans, and suicide attempts among university students in Uganda.

**Methods:**

Cross-sectional study data were collected from May to September 2021 from 540 undergraduate university students in south-western Uganda (363 males, mean age 23.3 years). Questions from the General Health Questionnaire (GHQ-28) were used to assess suicidal ideation, while other bespoke questions were used to assess suicide plans and attempts. The survey also investigated the suicide attempt/plan method, location of the suicidal activity, and reason for not enacting the suicide plan. Three independent regression analyses were used to determine the factors associated with different forms of suicidal behaviours.

**Results:**

The prevalence of past-year suicidal behaviours was 31.85% for suicidal ideation, 8.15% for suicide plans, and 6.11% for suicide attempts. Having a chronic physical medical condition increased the likelihood of having all forms of suicidal behaviours. Suicidal ideation was associated with having difficulty paying university tuition fees. However, being in the fifth year of university education, and feeling satisfied with current academic grades reduced the likelihood of suicidal ideation. Individuals feeling satisfied with academic performance appeared to be a protective factor against having suicide plans. Suicide attempts were associated with having a history of sexual abuse and having difficulty paying university tuition fees. The most common method used for attempted suicide was a drug overdose, and the most common location for attempted suicide was their homes.

**Conclusion:**

University students have prevalent suicide behaviours especially among students with a chronic physical medical condition, a history of sexual abuse, and problems paying university tuition fees. Based on the present study, for students at risk, universities should provide appropriate interventions such as life skills education and suicide prevention techniques.

**Supplementary Information:**

The online version contains supplementary material available at 10.1186/s12888-022-03858-7.

## Introduction

One in every 100 deaths globally is due to suicide [1]. The African region has a higher suicide rate (12.0 per 100,000) than the global average (10.5 per 100,000), and has the third highest suicide rate following Europe and South-East Asia [2]. In Uganda, the suicide rate was 4.6 per 100,000 people in 2018 [3], and many university students die by suicide [4, 5]. Suicide is the second leading cause of death among those aged 15 to 29 years, the age group in which most undergraduate university students are found [2]. Suicidal behaviours are common among university students with pooled prevalence rates of 22.3% for suicidal ideation, 6.1% for suicide plans, and 3.2% for suicide attempts [6].

Various factors have been identified as being associated with suicidal behaviours among university students including dissatisfaction with academic performance, mental health illness, physical illness, sexual orientation, having a history of drug abuse and alcohol use, poor relationship with parents, involvement in physical fights, history of sexual abuse, previous suicide attempts, financial stress, hopelessness, younger age, female gender, being underweight or overweight, personality disorders (e.g., borderline personality disorder), depression, problematic internet use, problem gambling, and social isolation [7–13]. In addition, the negative impact of social interaction among university students such as social events (e.g., clubbing) may lead to sleep problems and self-care deficits, as well as disengagement from hobbies, reading, organized religious activity, and exercise [7–10, 14, 15]. The COVID-19 pandemic and associated stressors have also been associated with an increase in suicidal behaviours among university students especially following the lockdown of academic institutes to reduce the spread of the infection [14]. Risk factors related to suicidal behaviours and suicide during the COVID-19 pandemic have included: increased alcohol and substance use, fear of infection, increased financial problems, travel restrictions preventing individuals seeing their loved ones in person, being suspected of having COVID-19, online schooling, and increased psychopathological stressors such as depression [16–22]. In Uganda, following the first case of COVID-19 in the country on March 21, 2020, all academic institutions were closed and students were confined in their homes to reduce spread of COVID-19 [23]. This was a period of heightened stress, anxiety, and depression among university students, and some students died by suicide [4, 24, 25].

Despite the various known risk factors, the efficacy of interventions targeting suicide prevention among university students is still inconclusive [26, 27]. In addition, there are inadequate methods to detect suicidal behaviours by many university health facilities due to poor mental health services within universities [27]. Nevertheless, recognition of suicide behaviours among students and their associated factors can facilitate the formulation of preventive measures, timely interventions, and better control of the problem. Moreover, based on the media reporting suicide, university students in Uganda have been reported to die by suicide (23 in a 10-year period from 2010 to 2020) [14]. There are also known suicide risk factors among Ugandan university students that have been reported during the COVID-19 pandemic including depression, burnout, anxiety, and stress [4, 24, 28, 29]. However, no previous multi-centre study has investigated suicidal behaviours in university students in Uganda. The lack of such data is a critical gap in literature. With evolving educational environments and ever emerging global health threats such as the COVID-19 pandemic, knowledge is required concerning suicidal behaviours of university students to ensure that wellbeing of students remains in check given the growing changes in the educational systems. Based on these aforementioned findings, the present study aimed to examine the prevalence and factors associated with suicidal behaviours among university students in south-western Uganda during the COVID-19 pandemic.

## Methods

### Study design and setting

The present cross-sectional study collected data utilizing an online survey. Student participants were recruited utilizing convenience sampling at four universities in south-western Uganda [Mbarara University of Science and Technology (MUST), Kabale University (KU), Kampala International University-Western Uganda Campus (KIU), and Bishop Stuart University (BSU)] during the academic year 2020/2021. MUST is a public university in south-western Uganda, Mbarara district, with two campuses and six faculties. It has a population of approximately 4260 students. KU is a public university, located in Kabale district with two campuses and seven faculties in extreme south-western Uganda. It has approximately 3000 students. KIU is a private university with nine faculties in western Uganda – Bushenyi district. It has approximately 17,000 students. BSU is a private based, not-for-profit chartered university with two campuses and five faculties and is located in Mbarara city in south-western Uganda. It has approximately 5800 students.

### Study sample size estimation

The minimum sample size required to produce statistical power of 80% was calculated using Epi Info StatCalc for Population Surveys (Version 7.2.2.6) using a population size of approximately 30,000 undergraduate university students, expected frequency of suicide behaviours at 50% (because no recent study concerning suicidal behaviours has been carried out among university students in Uganda, and a value that maximises sample size was used) [30], an acceptable margin of error of 5% and a design effect of 1.0. The minimum calculated sample size was 380. Using representative ratios from the different universities obtained by dividing the number of students per university by the total number of students, the minimum number of students for MUST = 64, BSU = 64, KU = 38 and KIU = 216.

### Data collection

The survey was carried out from May to September 2021, using a pretested survey hosted on *Google Forms*. Students have different closed *WhatsApp* groups, *Facebook* groups, group emails, and other online socializing groups, where the online survey link for data collection was distributed. The research team circulated the online survey link within the faculty and student social media platform networks. Using the following message: *“Dear all, I hope this finds you well. You are requested to fill out this questionnaire; it will take you only 5–10 min. This will help assess and help our friends who may be having suicide behaviours at campus. Your responses are all anonymous, confidential, and well protected. Your contribution is highly valued. Thank you for your participation.”*

A total of 1000 students were approached directly with weekly reminders through *WhatsApp* (250 per university), 540 students accepted to participate and were included in the final analysis (54% response rate).

### Study measures

The online survey consisted of questions relating to (i) socio-demographics, (ii) academic information, (iii) behavioural factors, and (iv) suicidal behaviours (ideations, plans, and attempts).

#### Socio-demographics and other factors

Socio-demographic information was collected including age (in years), gender (male or female), marital status (single, married/cohabiting, and separated/divorced), religion (Muslim, Christian, other), and places of residence at the university (university hall, rented house/room, hostel, or home). Academic information was also collected relating to program and year of study, university tuition fee sponsor (government, private, loan scheme, non-government organisation [NGO], or others), payment of university tuition fees, and satisfaction with current academic grades, as well as health factors such as history of mental illness and chronic physical medical conditions (e.g., asthma, diabetes, hypertension, HIV, etc.). Other information collected included substance use (cigarette/marijuana smoking, alcohol drinking), presence of relationship stress – problems from a romantic relationship that lead to stress, and sexual/physical abuse history all responded to dichotomously (i.e., ‘yes/no’).

#### Suicide behaviours

##### Suicidal ideation

Suicidal ideation was assessed using four questions concerning suicide from the 28-item General Health Questionnaire [31, 32]. The questions included: (1) “*In the past 12 months, have you felt that life is not worth living?*” (2) “*In the past 12 months, have you found yourself wishing you were dead and away from it all?*” (3) “*In the past 12 months, have you had thoughts of the possibility that you might do away with yourself?*”, and (4) “*In the past 12 months, have you found the idea of taking your own life kept coming into your mind?*”. These four questions are rated on a four-point scale where 0 = ‘not at all/definitely not’, 1 = ‘no more than usual/I don’t think so’, 2 = ‘rather more than usual/has crossed my mind’, and 3 = ‘much more than usual/definitely has’. A score of 2 or 3 was considered a positive response for suicidal ideation, whereas 0 or 1 was considered negative. A negative score was coded 0 and a positive score was coded 1. The binary scores for each of the four questions were added to give a zero to four score for suicidal ideation (continuous scale). A score of 1 and above was considered positive for suicidal ideation (giving a binary score for suicidal ideation).

##### Suicide plans

Suicide plans were assessed based on a question adopted from a study by Cheug et al. (2016) [33]. “*In the past 12 months, have you planned suicide?* (yes/no)”. If the participant had positive response, four follow-up questions were asked: (1) “*In the past 12 months, how frequently have you planned to commit suicide or take your own life?*” (response: rarely, once every month, once every week, every day, and always); (2) “*Which method(s) did you plan to use*?”; (3) “*Where were you planning to kill yourself?*”; and (4) “*What kept you alive and not proceeding with your plan?*”.

##### Suicide attempts

Participants were assessed for suicide attempts based on a question adopted from a study by Cheug et al. (2016) [33]. “*In the past 12 months, have you attempted to commit suicide or take your own life?*” (response: yes/no). If the participant responded “yes”, five follow-up questions were asked: (1) “*In the past 12 months, how frequently have you attempted to commit suicide or take your own life?*” (response: rarely, once every month, once every week, every day, or more than once a day); (2) “*In the past 12 months, did you need treatment as a result of attempting suicide?*” (response: yes/no); (3) *“Which method did you use to attempt suicide?*”; (4) “*Where did you attempt to kill yourself?*”; and (5) *“Did you write a suicide note”* (response: yes/no).

### Ethics

The present study was conducted in accordance with the Declaration of Helsinki 2013 [34]. The present study’s formal ethical approval was obtained from the Mbarara University of Science and Technology research ethics committee (MUSTREC #16/02–21). Permission to collect data from participants was granted by the Dean of Students at each of the four universities. All participants provided voluntary written informed consent at study enrolment. Participants were informed that they have a right to avoid responding to questions that trigger painful emotions. In case of distress or agitation, the participant was free to end the survey. In case they needed help, a link was provided within the data collection tool to reach the psychiatry team for psychological management.

### Statistical analysis

A *Google Forms* sheet with the captured data was imported into, cleaned, and analysed using the statistical software, STATA Version 16. Descriptive statistics (e.g., percentages, frequencies, means, and standard deviations) were used to analyse the data. Additionally, the Gaussian assumption was used to assess for normality based on the Shapiro-Wilks test and histograms. The reasons for not enacting suicide plans were analysed using frequencies and percentages of the responses. Separate regression analyses were used to determine the factors associated with suicide behaviours (suicidal ideation, suicide plans, and suicide attempts). Logistic regression analyses were used for suicidal ideation, suicide plans, and suicide attempts. All significant factors at bivariate regression were taken into the adjusted model to adjust for confounding variables following testing for collinearity based on variance inflation factor (VIF). Factors with a VIF of less than 3 were included in the final models. Model sensitivity, specificity, correctly classified for suicidal behaviours, and goodness of fit were calculated. A *p*-value of less than 0.05 was considered significant.

## Results

### Participant characteristics

The age of the participants ranged from 18 to 40 years, with a mean age of 23.3 years (SD ± 2.64). The majority of the participants were male (67.2%), and MUST had the highest number of students represented (51.5%). A total of 27.0% of the participants drank alcohol, while only 2.0% smoked cigarettes/marijuana. A total of 6.5% reported having had a mental health illness (see Table 1).

**Table 1:** Sociodemographic, clinical, and epidemiological characteristics of respondents (*n* = 540)

Variable	n (%)
**Total**	540 (100)
**Age** (µ ± *SD)*	23.3 (2.64)
**Sex**	
Female	177 (32.78)
Male	363 (67.22)
**Current university**	
BSU	59 (10.93)
KIU	127 (23.52)
MUST	278 (51.48)
KU	76 (14.07)
**Religion**	
Christian	500 (92.59)
Moslem	35 (6.48)
None	5 (0.93)
**Sponsor for paying university tuition fees**	
Government	107 (19.81)
Loan scheme	96 (17.78)
NGO	18 (3.33)
Private	288 (53.33)
Others	31 (5.74)
**Area of residence**	
Home	35 (6.48)
Hostel	204 (37.78)
Rentals	250 (46.30)
University hall	45 (8.33)
Others	6 (1.11)
**Marital status**	
Single	497 (92.04)
Co-habiting	20 (3.70)
Married	23 (4.26)
**College/faculty**	
Agriculture and Environment Sciences	7 (1.30)
Business and Management Sciences	27 (5.00)
Computing and Information Science	10 (1.85)
Education and External Studies	43 (7.96)
Engineering, Designing, Art, and Technology	34 (6.30)
Health Sciences/Medicine	322 (59.63)
Humanities and Social Sciences	6 (1.11)
Law	11 (2.04)
Others	80 (14.81)
**Year of study**	
First	78 (14.44)
Second	165 (30.56)
Third	143 (26.48)
Fourth	96 (17.78)
Fifth	49 (9.07)
Sixth	9 (1.67)
**Smoking cigarette/ marijuana**	
No	529 (97.96)
Yes	11 (2.04)
**Drinking alcohol**	
No	394 (72.96)
Yes	146 (27.04)
**Had relationship issues**	
No	266 (49.26)
Yes	274 (50.74)
**Had trouble paying university tuition fees**	
No	334 (61.85)
Yes	206 (38.15)
**Satisfied with academic grades**	
No	278 (51.48)
Yes	262 (48.52)
**Been sexually abused**	
No	483 (89.44)
Yes	57 (10.56)
**Been involved in physical fighting**	
No	467 (86.48)
Yes	73 (13.52)
**Been managed for any mental health issues**	
No	505 (93.52)
Yes	35 (6.48)
**Had a chronic physical medical condition**	
No	506 (93.70)
Yes	34 (6.30)

### Suicide behaviours

#### Suicidal ideation

**Fig. 1:**
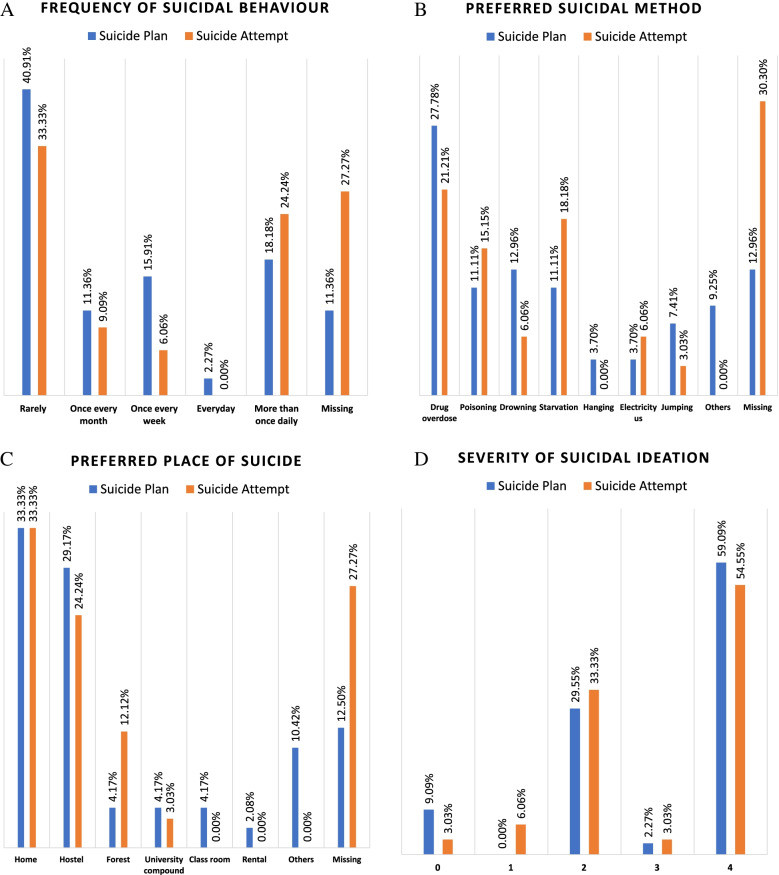
Characteristics of students who had a suicide plan and suicidal ideation

Suicidal ideation severity scores ranged from zero to four, with the majority of the students having no suicidal ideation (68.15%). The remainder scored 1 (12.22%), 2 (9.63%), 3 (2.22%), and 4 (7.78%). Therefore, using a binary score, 31.85% reported suicidal ideation. The majority of the individuals reporting severe suicidal ideation had suicide attempts and/or suicide plans **(**Fig. 1 and Supplementary Table 1).

#### Suicide plans

A total of 8.15% of the students had at least one suicide plan in the past 12 months, with one student thinking about suicide every day and the majority rarely thinking about suicide in the past 12 months (40.91%). However, five students did not respond concerning the frequency of their suicide plans. Drug overdose was the most commonly planned method (34.09%), followed by drowning (15.91%). The majority of the students planned to commit suicide at home (36.36%) followed by a hostel (31.82%) (see Fig. 1 and Supplementary Table 1).

#### Suicide attempts

The prevalence of suicide attempts in the past 12 months was 6.11%. One-third of these had rarely attempted suicide (33.33%). Drug overdose was the most common method for attempting suicide (21.21%, *n* = 7), followed by starvation, (18.18%, *n* = 6). One-third of the students attempted suicide in their homes (33.33%, *n* = 11). Only 9/33 suicide attempters (27.27%) required treatment following the suicide attempt, and four participants reported writing a suicide note before their suicide attempt (Fig. 1 and Supplementary Table 1).

### Reasons for not going through with the suicide

Two-thirds of students with suicide plans (*n* = 28) had reasons for not executing their plans (68.18%). This included religious beliefs (*n* = 9; 30%), support from friends and family during the time when the participant had a suicide attempt (*n* = 3; 10.71%), and fear of disappointing or leaving family and friends in agony (*n* = 5; 17.86). A quarter of the students decided they had hope for a better future (*n* = 8; 26.67%). Two students lacked time to execute their plans, and one student said they were extremely afraid to die.

### Factors associated with suicide behaviours

#### Suicidal ideation

Table 2 shows the bivariate analysis for factors associated with suicidal ideations, and they included the following: being of younger age, studying a subject in the Computing and Information Science faculty, having a history of having relationship issues, having trouble paying university tuition fees, not being satisfied with academic grades, having a history of being sexually abused, having a history of being treated for a mental health condition, and having a chronic physical medical condition. However, being in the fourth or fifth year of study was less associated with having suicidal ideation. These were tested for collinearity, and all had VIFs below 3, with a mean VIF of 1.12. They were used in building the final model using the backward stepwise selection method. The model had a sensitivity of 33.72%, specificity of 93.21%, PPV of 69.88%, NPV of 75.05%, and correctly classified 74.26% of all suicidal ideation. The goodness-of-fit *p*-value was 0.100 for the included nine variables. In the multivariate analysis, being in the fifth year of university education and satisfaction with academic performance reduced the likelihood of having suicidal ideation, (AOR = 0.21, 95% CI = 0.05–0.80, *p* = 0.023) and (AOR = 0.47, 95% CI = 0.31 – 0.72, *p* = 0.001), respectively. However, having trouble paying university tuition fees (AOR = 2.04, 95% CI = 1.34–3.12, *p* = 0.001) and having a chronic physical medical condition (AOR = 2.30, 95% CI = 1.01–5.25, *p* = 0.047) increased the likelihood of having suicidal ideation (see Table 3).

**Table 2:** Bivariate logistic regression for factors associated with suicide behaviours among university students in south-western Uganda. (*n* = 540)

Variable	Suicidal ideation			Suicide plans		Suicide attempts	
	**cOR (CI)**	***p*-value**		**cOR (CI)**	***p*-value**	**cOR (CI)**	***p*-value**
Age	0.88 (0.81 – 0.96)	**0.004**		0.96 (0.84 – 1.09)	0.544	0.91 (0.77 – 1.08)	0.277
**Sex**							
Female	1			1		1	
Male	0.72 (0.49 – 1.05)	0.090		0.84 (0.44 – 1.60)	0.597	0.74 (0.36 – 1.52)	0.405
**Current university**							
BSU	1		1			1	
KIU	0.57 (0.30 – 1.10)	0.094	0.59 (0.20 – 1.80)		0.356	0.30 (0.09 – 1.00)	0.051
MUST	0.67 (0.37 – 1.19)	0.172	0.65 (0.25 – 1.70)		0.378	0.42 (0.16 – 1.09)	0.075
KU	1.14 (0.57 – 2.28)	0.714	1.49 (0.52 – 4.31)		0.457	0.64 (0.20 – 2.01)	0.441
**Religion**							
Christian	1		1			1	
Muslim	0.97 (0.47 – 2.04)	0.944	1.05 (0.31 – 3.58)		0.938	1.47 (0.43 – 5.07)	0.543
None	0.53 (0.06 – 4.79)	0.573	Omitted			Omitted	
**Sponsor for paying university tuition fees**							
Government	1		1			1	
Loan scheme	1.48 (0.81 – 2.70)	0.204	1.72 (0.47 – 6.26)		0.414	1.12 (0.27 – 4.60)	0.876
NGO	1.80 (0.63 – 5.09)	0.271	5.15 (1.05 – 25.30)		**0.044**	3.22 (0.54 – 19.03)	0.197
Private	1.35 (0.82 – 2.21)	0.242	2.66 (0.91 – 7.80)		0.074	1.92 (0.64 – 5.76)	0.243
Other	1.55 (0.66 – 3.64)	0.313	3.81 (0.90 – 16.25)		0.070	2.76 (0.58 – 13.05)	0.201
**Type of residence**							
Home	1		1			1	
Hostel	0.74 (0.35 – 1.59)	0.445	0.67 (0.18 – 2.49)		0.547	0.49 (0.13 – 1.92)	0.307
Rentals	1.02 (0.49 – 2.15)	0.952	1.29 (0.37 – 4.50)		0.688	0.88 (0.25 (3.13)	0.840
University hall	0.96 (0.38 – 2.44)	0.929	0.50 (0.08 -3.15)		0.457	0.50 (0.08 – 3.15)	0.457
Other	0.38 (0.04 – 3.66)	0.405	Omitted			Omitted	
**Marital status**							
Single	1		1			1	
Co-habiting	0.70 (0.25 – 1.95)	0.490	1.30 (0.29 – 5.83)		0.728	Omitted	
Married	0.74 (0.29 – 1.90)	0.528	1.76 (0.50 – 6.19)		0.377	1.43 (0.32 – 6.39)	0.638
**College/Faculty**							
Agriculture and Environment Sciences	1		1			1	
Business and Management Sciences	2.69 (0.44 – 16.37)	0.282	0.75 (0.66 – 8.55)		0.817	1.04 (0.10 – 11.14)	0.972
Computing and Information Science	10.00 (1.05 – 95.46)	**0.045**	1.50 (0.11 – 20.68)		0.762	0.67 (0.03 – 12.84)	0.788
Education and External Studies	2.17 (0.38 – 12.46)	0.383	1.17 (0.12 – 11.25)		0.894	0.45 (0.04 – 5.06)	0.518
Engineering, Designing, Art, and Technology	1.97 (0.33 – 11.63)	0.453	0.80 (0.08 – 8.47)		0.853	0.58 (0.05 – 6.57)	0.661
Health Sciences/Medicine	0.80 (0.15 – 4.20)	0.791	0.38 (0.04 – 3.29)		0.377	0.23 (0.03 – 2.08)	0.192
Humanities and Social Sciences	2.50 (0.25 – 24.72)	0.433	1.20 (0.06 – 25.47)		0.906	1.20 (0.06 -24.47)	0.906
Law	1.43 (0.18 – 11.09)	0.733	Omitted			Omitted	
Others	1.35 (0.25 – 7.39)	0.732	0.58 (0.06 – 5.48)		0.631	0.67 (0.71 – 6.26)	0.723
**Year of study**							
First	1		1			1	
Second	0.74 (0.43 – 1.27)	0.277	0.63 (0.27 – 1.49)		0.293	0.49 (0.20 – 1.20)	0.117
Third	0.61 (0.35 – 1.07)	0.084	0.57 (0.23 – 1.40)		0.219	0.30 (0.10 – 0.85)	**0.024**
Fourth	0.29 (0.15 – 0.56)	** < 0.001**	0.53 (0.19 – 1.48)		0.227	0.45 (0.16 – 1.31)	0.144
Fifth	0.08 (0.02 – 0.27)	** < 0.001**	0.14 (0.02 – 1.14)		0.067	Omitted	
Sixth	0.15 (0.02 – 1.22)	0.076	0.85 (0.10 – 7.54)		0.884	Omitted	
**Smoking cigarette/marijuana**							
No	1		1			1	
Yes	1.81 (0.54 – 6.00)	0.334	4.46 (1.14 – 17.47)		**0.032**	3.57 (0.74 – 17.24)	0.113
**Drinking alcohol**							
No	1		1			1	
Yes	1.11 (0.74 – 1.67)	0.604	0.89 (0.44 – 1.81)		0.751	1.19 (0.55 – 2.56)	0.663
**Having relationship issues**							
No	1		1			1	
Yes	1.60 (1.11 – 2.31)	**0.011**	0.88 (0.47 – 1.63)		0.677	1.03 (0.51 – 2.09)	0.927
**Had trouble paying university tuition fees**							
No	1		1			1	
Yes	2.47 (1.70 3.58)	** < 0.001**	2.54 (2.35 – 4.75)		**0.004**	3.51 (1.66 – 7.40)	**0.001**
**Satisfied with academic performance**							
No	1		1			1	
Yes	0.46 (0.31 – 0.66)	** < 0.001**	0.42 (0.21 – 0.81)		**0.010**	0.77 (0.38 – 1.57)	0.471
**Been sexually abused**							
No	1		1			1	
Yes	2.10 (1.20 – 3.65)	**0.009**	3.26 (1.55 – 6.88)		**0.002**	3.59 (1.58 – 8.16)	**0.002**
**Been involved in physical fighting**							
No	1		1			1	
Yes	1.22 (0.72 – 2.04)	0.458	1.01 (0.41 – 2.48)		0.981	1.15 (0.43 – 3.09)	0.777
**Been treated for any mental health condition**							
No	1		1			1	
Yes	3.10 (1.54 – 6.21)	**0.001**	3.16 (1.29 – 7.73)		**0.012**	2.84 (1.02 – 7.88)	**0.045**
**Having chronic physical medical condition**							
No	1		1			1	
Yes	3.80 (1.85 – 7.78)	** < 0.001**	4.02 (1.70 – 9.51)		**0.002**	5.92 (2.43 – 14.40)	** < 0.001**

**Table 3:** Multivariate logistic regression for factors associated with suicide behaviours among university students in south-western Uganda

Variable	Suicidal ideation		Suicide plans		Suicide attempt	
	**cOR (CI)**	***p*-value**	**cOR (CI)**	***p*-value**	**cOR (CI)**	***p*-value**
Age	0.94 (0.86 – 1.03)	0.210				
**Sponsor for paying university tuition fees**						
Government			1			
Loan scheme			1.65 (0.43 – 6.31)	0.461		
NGO			3.95 (0.74 – 21.19)	0.109		
Private			2.04 (0.65 – 6.46)	0.224		
Others			3.33 (0.72 – 15.45)	0.124		
**College/Faculty**						
Agriculture and Environment Sciences	1					
Business and Management Sciences	2.20 (0.33 – 14.57)	0.413				
Computing and Information Science	7.53 (0.71 – 79.48)	0.093				
Education and External Studies	1.48 (0.24 – 9.17)	0.671				
Engineering, Designing, Art, and Technology	1.33 (0.21 – 8.51)	0.766				
Health Sciences/Medicine	0.82 (0.15 – 4.57)	0.817				
Humanities and Social Sciences	1.00 (0.09 – 11.24)	0.997				
Law	1.05 (0.12 – 9.06)	0.963				
Others	0.81 (0.14 – 4.81)	0.821				
**Year of study**						
First	1				1	
Second	0.97 (0.53- 1.81)	0.934			0.61 (0.23 – 1.61)	0.314
Third	0.88 (0.46 – 1.69)	0.708			0.41 (0.13 – 1.29)	0.127
Fourth	0.56 (0.26 – 1.21)	0.141			0.80 (0.25 – 2.57)	0.713
Fifth	0.21 (0.05 – 0.80)	**0.023**			Omitted	
Sixth	0.32 (0.03 – 3.10)	0.327			Omitted	
**Smoking cigarette/marijuana**						
No			1			
Yes			2.78 (0.52 – 14.99)	0.235		
**Having relationship issues**						
No	1					
Yes	1.24 (0.83 – 1.87)	0.298				
**Had trouble paying university tuition fees**						
No	1		1		1	
Yes	2.04 (1.34 – 3.12)	**0.001**	1.75 (0.87 – 3.54)	0.116	2.51 (1.13 – 5.55)	**0.023**
**Satisfied with academic grades**						
No	1		1			
Yes	0.47 (0.31 – 0.72)	**0.001**	0.46 (0.23 – 0.93)	**0.030**		
**Been sexually abused**						
No	1		1		1	
Yes	1.61 (0.82 – 3.16)	0.163	2.13 (0.90 – 5.04)	0.085	3.02 (1.21 – 7.51)	**0.017**
**Been treated for any mental health condition**						
No	1		1		1	
Yes	1.71 (0.74 – 3.97)	0.212	1.71 (0.60 – 4.87)	0.312	1.08 (0.32 – 3.63)	0.903
**Having a chronic physical medical condition**						
No	1		1		1	
Yes	2.30 (1.01 – 5.25)	**0.047**	3.08 (1.14 – 8.28)	**0.026**	3.93 (1.48 – 10.46)	**0.006**

#### Suicide plans

Collinearity was established among the following variables: having university tuition sponsored by an NGO, smoking cigarettes or marijuana, having trouble paying university tuition fees, non-satisfaction with academic performance, history of sexual abuse, having a history of being treated for a mental health condition, and having a chronic physical medical condition (Table 2) with a mean VIF of 1.10 and all VIFs were below 3. These variables were included in the final multivariate model and having a chronic illness increased the likelihood of having suicide plans (AOR = 3.08, 95% CI = 1.14–8.28; *p* = 0.026) while being satisfied with academic grades lowered the likelihood of having suicide plans (AOR = 0.46, 95% CI = 0.23–0.93, *p* = 0.030). For details, see Table 3. The model had a sensitivity of 4.55%, specificity of 100.00%, PPV of 100.00%, NPV of 92.19%, and correctly classified 92.22% of having suicide plans. The goodness-of-fit *p*-value was 0.080.

#### Suicide attempts

The factors that were significant in the bivariate analysis for suicide attempts included being in the third year of study, having trouble paying university tuition fees, having a history of sexual abuse, having a history of being treated for a mental health condition, and having a chronic physical medical condition (Table 2). They had a mean VIF of 1.08, and all were below VIF of 3. In the multivariate analysis, the likelihood of suicide attempt was highest with having a chronic physical medical condition (AOR = 3.93, 95% CI = 1.48–10.46, *p* = 0.006), followed by having history of being sexually abused (AOR = 3.02, 95% CI = 1.21–7.51, *p* = 0.017), and lowest if a student had trouble paying university tuition fees (AOR = 2.51, 95% CI = 1.13–5.55, *p*-value = 0.023) (see Table 3). The model had a sensitivity of 6.06%, specificity of 100.00%, PPV of 100.00%, NPV of 93.54%, and correctly classified 93.57% of having a suicide attempt. The goodness-of-fit *p*-value was 0.445.

## Discussion

The present study determined the prevalence of suicidal ideation, suicide plans, and suicide attempts, as well as the associated factors among university students in south-western Uganda. The prevalence of past-year suicidal behaviours was 31.85% for suicidal ideation, 8.15% for suicide plans, and 6.11% for suicide attempts. Having a chronic physical medical condition increased the likelihood of having all forms of suicide behaviours. However, having trouble paying university tuition fees increased the likelihood of suicidal ideation. Satisfaction with academic performance was protective against having suicide plans. Among those reporting suicide attempts, having a history of sexual abuse and university tuition constraints increased the likelihood. The most attempted suicide method was a drug overdose, with most preferring to attempt suicide in their homes.

To date, only one study [35] has assessed suicidal behaviours among university students in Uganda (2000–2003). The reported prevalence of suicidal ideation was 56% among non-medical students at Makerere University before introducing peer counselling services (2000–2001) and was higher than that reported in the present study. However, following the introduction of peer counselling services in 2002, the prevalence of suicidal ideation significantly reduced to 8.9% and is lower than that reported in the present study. Currently, counselling services are present in all universities in Uganda but these services were not present at university during the lockdown period during the COVID-19 pandemic. Home confinement during the pandemic and the lack of face-to-face emotional support at universities was unavailable and may have increased the incidence of suicide behaviours during this period. The large difference between the two studies following the introduction of peer counselling services introduction may be because: (i) the present study was conducted during the COVID-19 pandemic – a period characterized with multiple stressors and increase in suicidal behaviours [14, 36]; (ii) of the time period between the studies, the former being conducted during a period with lesser mental stressors such as internet use disorders and lower mental health problem prevalence rates, and (iii) the two studies were conducted using different tools for accessing suicidality (Response Inventory for Stressful Life Events vs. four items from the GHQ-28), which may have caused the difference since the two instruments have never been compared directly in terms of their psychometric properties.

The suicide behaviours among undergraduate students in the present study were still higher than those in a study among that those reported from a study done in a similar setting (i.e., Ghana) in 2020, where the prevalence rates were 15.2% for suicidal ideation, 6.8% for suicide plans, and 6.3% for suicide attempts [37]. However, the prevalence rates were lower than that among 122 psychology undergraduate students in Botswana, which reported 47.5% for suicidal ideation (based on Beck’s Depression Inventory Item 9), and 28.7% for suicide attempts [38]. However, the study only involved students from one institute and the sample size was very small, which could have influenced the reported prevalence rates. In comparison with some studies from high-income countries and other parts of the world conducted during the pandemic, the present study’s reported prevalence rates were higher [39–42].

Most of the participants in the present study reporting suicide plans and attempts reported the use of a drug overdose to end their lives. This is not surprising since the dominant activity in south-western Uganda is farming, where suicide agents such as herbicides and pesticides are commonly used [43]. Among the same group, the majority planned or attempted suicide from their homes, possibly because they had easy access to these poisonous agents. This finding is similar to the other study from Uganda, which reported that university students preferred to die in their homes [4]. Of the students that had attempted suicide, only a quarter of them wrote a suicide note. This is lower than that reported in a study in Botswana where nearly half of the students reporting suicide plans or attempts had written a suicide note [38]. However, it is higher than the 0.4% among 6,838 students in China during the pandemic [40]. The higher rates in the African countries may be due to individuals trying to communicate their grievances and stressors to their loved ones since students do not find talking about their stressors easily [4]. As in previous studies, hope for the future, religion, social support, and fear of death were the strongest motivators for students deciding not to carry out suicide [44, 45].

Having a chronic physical medical condition was a common factor associated with all types of suicidal behaviours, and led to the highest likelihood of a suicide attempt. This has been a consistently reported risk factor for suicide within other populations [46], but no previous study has found it to be associated with suicide behaviours among university students. Suicide behaviours are higher among individuals with chronic physical medical conditions because of the level of progression of the disease, the presence of symptoms of depression, feelings of helplessness, disruptive interpersonal relationships, and uncontrolled pain [46, 47]. For university students, the stress levels brought on by the presence of a chronic physical medical condition are aggravated by stress due to being a university student, such as poor academic performance, tuition and sponsorship problems, and challenges associated with adjusting to university, especially in the first years of university education. These factors were also associated with suicidal behaviours in the present study.

At university, academic performance is a measure of the level of knowledge and is reported to be a predictor of future success. In Uganda, education is considered the ‘key to success’ [48], and parents and students have high expectations, making many students with poor grades feel hopeless about their future due to them being unsatisfied with their studies. Satisfaction with academic performance is a major protective factor against suicidal behaviours due to the hope of a positive future [49]. In addition, progress in education depends upon funding (i.e., being able to pay the tuition fees and scholarship). However, the majority of individuals at university in Uganda have university education tuition fees privately paid for (by families or self) and the families are poor and cannot afford the expensive university education. A minority of these may lose hope for the future and have suicidal behaviours, and in extreme cases, engage in suicide. The COVID-19 pandemic-related restrictions increased financial problems for university students in Uganda especially those who worked to earn money to pay for tuition fees and/or living costs. Common employment ventures for university students like riding motorcycle taxis (bodaboda) were stopped for over one year, leaving students with no source of funding and increasing their mental health suffering, which might have led to them to engaging in suicide behaviours [50].

In addition to the aforementioned factors associated with suicide behaviours, age has consistently been associated with suicide behaviours, i.e., younger individuals globally – especially adolescents – have a higher risk of suicide compared to older individuals [51]. This finding was echoed in the present study especially regarding suicidal ideation, with it being more severe among younger students. In addition, adverse childhood events such as sexual abuse (associated with suicide attempts in the present study) may be among other factors leading to the strong consistent association, with younger students still adjusting to the more recent trauma compared to older students making them more prone to suicide.

### Limitations

The findings of the present study have to be interpreted with caution due to a number of limitations. First, the study not rigorously assess the different chronic physical diseases to examine their specific effects on suicide behaviours. Second, the study was cross-sectional and causality between the variables cannot be determined. Thirdly, the study was conducted during the COVID-19 pandemic, a known risk factor for suicide behaviours due to its impact on most individuals' social, academic, occupational, and financial functioning. There is also a possibility that the reported chronic medical conditions could have been COVID-19 since the question did not specify the conditions and some students have no medical background. Fourth, there is no guarantee that all the participants were truly university students since the link was open to anyone who could access it. Fifth, the study had a modest response rate (54%) which might make it unrepresentative of the targeted group of students. It should also be noted that the sampling method (i.e., convenience sampling) means that the participants may have been unrepresentative of the target population. Future studies should use alternative methods to recruit students such as targeting them before attending a classroom, repeated reminders to those who get the survey link, and incentivizing students to participate by having a lottery draw or some other activity that comes with an immediate reward for participation. Finally, all the data were self-reported and such data are subject to individual biases (e.g., memory recall).

## Conclusions

The present study found that university students have prevalent suicide behaviours especially among students with chronic disease, history of sexual abuse, and problems with paying university tuition fees. Suicide behaviours decrease as students’ progress in university education. Therefore, universities should target prevention efforts among students such as freshers. Moreover, students in upper academic years should assist and mentor those in lower years and share university life-solving hacks such as dealing with poor academic grades through peer support systems (including social and emotional support through social media and other online platforms) to reduce suicide behaviours occurring among university students. In addition, universities should organise meetings to share strategies to reduce suicide behaviours among university students since some of the study programs offered in all the universities, in the present study (i.e., computing and information science) had students with lower likelihood of having suicide behaviours. Based on the present study findings, for students at risk, universities should provide appropriate interventions such as life skills education and suicide prevention techniques.

## Supplementary Information

**Additional file 1.**

## Data Availability

The datasets used and/or analysed during the present study are available from the corresponding author on reasonable request.
