# Supplementary material for: Suicidal behaviours among Ugandan university students: a cross-sectional study
Source: BMC Psychiatry. 2022 Apr 1;22:234. doi: 10.1186/s12888-022-03858-7 (PMC8972906; doi:10.1186/s12888-022-03858-7)
Supplement: Supplementary file 1 — Additional file 1. [file 12888_2022_3858_MOESM1_ESM.docx]

**Supplementary Table 1: Characteristics of students who had a suicide plan (n=44) or suicide attempt (n=33)**

| **Variable** | **Suicide plan;** 44 (8.15%) | **Suicide attempt;** 33 (6.11%) |
| --- | --- | --- |
| **Frequency of suicidal behaviour** | | |
| Rarely | 18 (40.91) | 11 (33.33) |
| Once every month | 5 (11.36) | 3 (9.09) |
| Once every week | 7 (15.91) | 2 (6.06) |
| Everyday | 1 (2.27) | 0 (0.00) |
| More than once a day | 8 (18.18) | 8 (24.24) |
| Missing | 5 (11.36) | 9 (27.27) |
| **Preferred suicidal method** | | |
| Drug overdose | 15 (34.09) | 7 (21.21) |
| Poisoning | 6 (13.64) | 5 (15.15) |
| Drowning | 7 (15.91) | 2 (6.06) |
| Starvation | 6 (13.64) | 6 (18.18) |
| Hanging | 2(4.50) | 0 (0.00) |
| Use of electricity | 2 (4.50) | 2 (6.06) |
| Jumping from a height | 4 (9.09) | 1 (3.03) |
| Suffocation | 1 (2.27) | 0 (0.00) |
| Cutting self | 1 (2.27) | 0 (0.00) |
| Throwing self in a speeding car | 1 (2.27) | 0 (0.00) |
| Moving to dangerous places to be killed by strangers | 1 (2.27) | 0 (0.00) |
| Being used for clinical trial to mask suicide | 1 (2.27) | 0 (0.00) |
| Missing | 7 (15.91) | 10 (30.30) |
| **Preferred place of suicide (location)** | | |
| Home | 16 (36.36) | 11 (33.33) |
| Hostel | 14 (31.81) | 8 (24.24) |
| Forest | 2 (4.50) | 4 (12.12) |
| University compound | 2 (4.50) | 1 (3.03) |
| Class room | 2 (4.50) | 0 (0.00) |
| Rental | 1 (2.27) | 0 (0.00) |
| Others | 5 (11.36) | 0 (0.00) |
| Missing | 6 (13.64) | 9 (27.27) |
| **Treatment received following suicide attempt** | | |
| No | NA | 24 (72.73) |
| Yes | NA | 9 (27.27) |
| **Wrote a suicide note** | | |
| No | NA | 29 (87.88) |
| Yes | NA | 4 (12.12) |
| **Severity of suicidal ideation** |  |  |
| 0 | 4 (9.09%) | 1 (3.03%) |
| 1 | +0 (0.00%) | 2(6.06%) |
| 2 | 13 (29.55%) | 11 (33.33%) |
| 3 | 1 (2.27%) | 1(3.03%) |
| 4 | 26 (59.09%) | 18 (54.55%) |
